# Symptomatic Colorectal Cancer Is Associated With Stage IV Diagnosis in Two Disparate Populations

**DOI:** 10.7759/cureus.28691

**Published:** 2022-09-02

**Authors:** Carmen Fong, Dimitri Joseph, Samuel Stanley, Yicong Zhu, Wei Zhu, Evan Grossman, Henry Talus, Maksim Agaronov, Alexandra Guillaume, Paula Denoya

**Affiliations:** 1 Surgery, Division of Colon and Rectal Surgery, Mount Sinai Hospital, New York, USA; 2 Gastroenterology and Hepatology, Stony Brook University Hospital, Stony Brook, USA; 3 Applied Mathematics & Statistics, Stony Brook University, Stony Brook, USA; 4 Gastroenterology and Hepatology, NYC Health and Hospitals/Kings County, Brooklyn, USA; 5 Colon and Rectal Surgery, State University of New York Downstate Health Sciences University, Brooklyn, USA; 6 Pathology, NYC Health and Hospitals/Kings County, Brooklyn, USA; 7 Surgery, Division of Colon and Rectal Surgery, Stony Brook University Hospital, Stony Brook, USA

**Keywords:** cancer, lower gi or colorectal surgery, colon cancer, colonoscopy surveillance, colonoscopy, screening, colon cancer prevention, racial disparity, healthcare inequality, health care disparities

## Abstract

In the United States, individuals of Black/African Ancestry (AA) have a higher incidence and mortality from colorectal cancer (CRC) compared to individuals of White/European Ancestry (EA). In order to develop an approach towards disentangling the complex effects of associated race and socioeconomic factors on CRC outcome, we have conducted a manual chart review of sporadic CRC pathological diagnoses (total n = 334) at an urban public hospital (UH) and a suburban university hospital (SH). There were significant differences between the SH and UH CRC patients with respect to Black/AA race (4.2% vs. 89.1%, p < 0.0001) and Medicaid/Self-pay insurance status (14.9% vs. 85.0%, p < 0.0001). While a higher proportion of newly diagnosed CRC patients presented with metastatic stage 4 CRC at the UH (21%) than the SH (12.5%), only the presence of symptoms was significantly associated with stage 4 CRC (odds ratio, OR 7.94, 95% confidence interval, CI 1.83- 34.54, p = 0.0057) in a multivariable generalized linear model (GLM). The proportion of asymptomatic CRC patients was ~20% at both institutions, suggesting that the UH has contributed to reducing CRC disparities. Initiation of CRC screening at the recommended age at both institutions could reduce the proportion of CRC patients presenting with metastatic spread.

## Introduction

Colorectal cancer (CRC) is the second leading cause of cancer deaths in the United States [1]. The incidence and mortality of CRC have steadily decreased over the past two decades. Increased participation in CRC screening programs, because colorectal polyps detected during screening colonoscopies can be removed before they can turn into cancers, or CRCs can be detected earlier at less advanced stages, have likely contributed to the decreased incidence. Nevertheless, the incidence and mortality for CRC in the United States remains higher in Black/African Ancestry (AA) than all other race/ethnicity groups [2]. Associated racial differences in insurance status may affect access to quality CRC screening procedures. Associated racial differences in the prevalence of smoking, obesity, and diabetes mellitus could also contribute to Black/AA CRC risk [3-6]. Understanding the contributions of these factors is important for designing the most effective approaches towards reducing CRC incidence and mortality risk in the Black/AA populations. 

We have previously adopted a global approach towards collecting potential covariates that may contribute to racial disparities, in a retrospective chart review of initial screening colonoscopies performed at these two hospitals. Initial chart review of procedures performed in 2012 revealed low average adenoma detection rates in screening colonoscopies performed by some operators at one institution [7]. After intense physician feedback, the average adenoma detection rates increased to above national levels at both institutions by 2017 [5]. 

We now present the results of analyzing initial sporadic CRC diagnosed at these two institutions based on a chart review of the CRC specimens archived in the pathology databases between (January 1, 2010 and December 31, 2018). Clinic and procedure notes were carefully reviewed to determine whether the CRC was diagnosed in a patient undergoing asymptomatic screening colonoscopy or a diagnostic procedure (colonoscopy, imaging guided biopsy, or surgery) in order to obtain the diagnostic pathology specimen. The primary outcome measured was the CRC stage at the time of initial presentation and pathological diagnosis. 

## Materials and methods

Collection of data

This is a retrospective cohort study performed at a suburban hospital (SH) and an urban hospital (UH). Patients with a pathological diagnosis of CRC were identified by a review of pathology and endoscopy databases at the two hospitals. Patients with a history of previous CRC now presenting with recurrent or metachronous tumor, inflammatory bowel diseases, or known hereditary CRC syndromes were excluded from the analysis. Clinical metadata were manually collected on each patient using electronic medical records (EMRs) at the two hospitals. Datapoints recorded included: age at the time of pathologic diagnosis of CRC; sex (Male, Female); race (White/EA, Black/AA, Asian, Other); Hispanic ethnicity; insurance (Commercial, Medicare, Medicaid, Self-pay); body mass index (BMI) (kg/m2); tobacco exposure (current within one year, past use greater than one year, never); Family history of first degree relative with CRC; History of screening colonoscopy prior to diagnosis (Age < 50 y, Age > 80 y, yes, no); Interval CRC defined as diagnosis sometime after a normal screening colonoscopy (yes, diagnosis made within recommended interval for repeat surveillance or screening colonoscopy; no; unknown); Anatomic location of CRC (right or transverse colon to cecum, left or splenic flexure to sigmoid colon, rectum); CRC stage at diagnosis (0-4); and Surgical resection (yes, no), as previously described [5, 7]. Patients were phenotyped as diabetic if this diagnosis was in the EMR or if a recent hemoglobin A1c (HbA1c) was ≥ 6.5% [8]. CRC patients were diagnosed as symptomatic at the time of diagnosis if the EMR recorded rectal bleeding, iron deficiency anemia, or abdominal pain prior to the colonoscopy.

Statistical analysis

Demographics were compared between the UH and the SH populations using either the Wilcoxon rank-sum test for continuous variables or the chi-square test with exact p-values from Monte Carlo simulations for categorical variables. For the combined UH and SH datasets, both a multivariable generalized linear model (GLM) were conducted, with the primary outcome being either metastatic CRC (stage 4) at diagnosis or resectable CRC (stages 0,1,2,3). There was also a significant amount of missing data for family history of first-degree relative CRC in the UH dataset. For these reasons, family history of a first-degree relative with CRC was not included in the multivariable GLM. Statistical analysis was performed using R, and the significance level was set at 0.05. 

## Results

A total of 267 sporadic CRC patients from the SH and 129 CRC patients from the UH were included in this retrospective analysis (Table 1). CRC patients with a previous history of CRC, inflammatory bowel disease, or hereditary CRC syndromes (e.g., Lynch syndrome), were excluded from the analysis. Because the family history of a first-degree relative of CRC was missing from 75 cases, this variable was not included in the analysis. The UH CRC subjects were predominantly Black/AA (89.1%) and underinsured (Medicaid/Self-pay, 85%), compared to the SH CRC subjects, who were predominantly non-Hispanic White/EA (89.9%) and insured with either commercial or Medicare insurance (85%). There were also significant differences in the percentage of never smokers and diabetes mellitus between SH and UH CRC patients.

**Table 1:** Comparison of CRC patient characteristics between UH and SH. UH, urban hospital; SH, suburban hospital; IQR, interquartile range; BMI, body mass index; CRC, colorectal cancer

Variable	Missing	Level	Total N = 396	SH N = 267	UH N = 129	p-value
Median age (y) ± IQR	0	267 vs. 129	64.0 ± 17.0	64.0 ± 19.0	63.0 ± 13.0	0.7568
Male sex (%)	0	Yes vs. No	232 (58.6%)	161 (60.3%)	71 (55.0 %)	0.3341
Race (%)	0	White/EA	241 (60.9%)	240 (89.9%)	1 (0.8%)	<0.0001>
		Black/AA	126 (31.8%)	11 (4.1%)	115 (89.1%)	
		Other	29 (7.3%)	16 (6.0%)	13 (10.1%)	
Hispanic ethnicity (%)	2	Yes vs. No	38 (9.6%)	32 (12.0%)	6 (4.7%)	0.0295
BMI kg/m^2^ ± IQR	1	267 vs. 128	27.1 ± 7.5	27.6 ± 7.4	26.5 ± 6.5	0.0616
Diabetes mellitus (%)	0	Yes vs. No	116 (29.3%)	65 (24.3%)	51 (39.5%)	0.0017
Smoking (%)	11	Current	54 (14.0%)	36 (13.5%)	18 (15.3%)	<0.0001>
		Former	143 (37.1%)	121 (45.3%)	22 (18.6%)	
		Never	188 (48.8%)	110 (41.2%)	78 (66.1%)	
Insurance (%)	1	Commercial	152 (38.5%)	129 (48.3%)	23 (8.0%)	<0.0001>
		Medicare	107 (27.1%)	98 (36.7%)	9 (7.0%)	
		Medicaid	92 (23.3%)	3 (11.2%)	62 (48.4%)	
		Self-pay	44 (11.1%)	10 (3.7%)	34 (26.6%)	

As shown in Table 2, 334 patients presented with resectable CRC stage (Stages 0-3) and 64 with metastatic CRC stage (Stage 4) in the combined SH and UH dataset. Univariate analysis revealed significant associations of insurance (p = 0.0127), previous colonoscopy, symptoms at diagnosis, and institution, with Stage 4 CRC. Anatomic location was included in the analysis because rectal and distal left-sided lesions are more likely to present with visible rectal bleeding than right-sided lesions [9]. However, the association of anatomic location with stage 4 CRC did not reach significance.

**Table 2:** Univariate analysis of the effect of variables on CRC stage at diagnosis. SH, suburban hospital; UH, urban hospital; EA, European ancestry; AA, African ancestry; CRC, colorectal cancer

Variable	Missing	Level	Total N = 396	Resectable CRC Stage 0-3 N = 334	Metastatic CRC Stage 4 N = 62	p-value
Age (y) ± IQR	0	334 vs. 62	64.0 ± 17.0	64 ± 17.5	61.5 ± 14.8	0.0720
Male sex (%)	0	Yes vs. No	232 (58.6%)	194 (58.1%)	38 (61.3%)	0.6813
Race (%)	0	White/EA	241 (60.9%)	211 (63.2%)	30 (48.4%)	0.0917
		Black/AA	126 (31.8%)	100 (29.9%)	26 (41.9%)	
		Other	29 (7.3%)	23 (6.9%)	6 (9.7%)	
Hispanic ethnicity (%)	2	Yes vs. No	38 (9.6%)	30 (9.0%)	8 (13.1%)	0.3439
BMI (kg/m^2^) ± IQR	1	333 vs. 61	27.1 ± 7.5	27.4 ± 7.2	25.5 ± 7.9	0.4647
Diabetes mellitus (%)	0	Yes vs. No	116 (29.3%)	101 (30.2%)	15 (24.2%)	0.3666
Smoking (%)	11	Current	54 (14.0%)	44 (13.5%)	10 (16.9%)	0.7365
		Former	143 (37.1%)	123 (37.7%)	20 (33.9%)	
		Never	188 (48.8%)	159 (48.8%)	29 (49.2%)	
Insurance (%)	1	Commercial	152 (38.5%)	124 (37.2%)	28 (45.2%)	0.0126
		Medicare	107 (27.1%)	99 (29.7%)	8 (27.1%)	
		Medicaid	92 (23.3%)	78 (23.4%)	14 (23.3%)	
		Self-pay	44 (11.1%)	32 (9.6%)	12 (11.1%)	
Symptoms (%)	6	Yes vs. No	317 (81.3%)	259 (78.7%)	58 (95.1%)	0.0034
Previous colonoscopy age 50-80 y (%)	25	Age < 50 y	42 (11.3%)	32 (10.2%)	10 (17.5%)	0.0139
		Yes, age 50-80 y	63 (17.0%)	58 (18.5%)	5 (8.8%)	
		No, age 50-80 y	221 (59.6%)	181 (57.6%)	40 (70.2%)	
		Age > 80 y	45 (12.1%)	43 (13.7%)	2 (3.5%)	
CRC location (%) *	0	Right	164 (41.4%)	143 (42.8%)	21 (33.9%)	0.1724
		Left	144 (36.4%)	115 (34.4%)	29 (46.8%)	
		Rectum	90 (22.7%)	78 (23.3%)	12 (19.4%)	
Institution (%)	0	SH	267 (67.4%)	233 (69.8%)	34 (54.8%)	0.0287
		UH	129 (32.6%)	101 (30.2%)	28 (45.2%)	

In a multivariable GLM (Table 3), only the presence of symptoms at diagnosis was significantly associated (OR 8.04, 95% CI 1.85-35.01, p = 0.0055) with stage 4 CRC. This model included the variables in Table 2 with p < 0.1, and excluded subjects aged <50 y and > 80 y, who would have been outside United States Preventive Services Task Force (USPSTF) recommended age of initiating CRC screening (age 50 y during 2010-2018) and at least 5 y beyond the upper age (75 y) for continuing routine CRC screening. In this model, the total sample size was 294 after the removal of CRC patients < 50 y and > 80 y of age. This is because subjects aged <50 or > 80 y, who were outside the USPSTF recommended age range (50-75 y) for routine CRC screening during 2010-2018, were excluded (USPSTF, 2021). None of the other variables were significantly associated with stage 4 CRC, after the removal of the symptoms variable from the model. Of note, there was no significant difference in the proportion of CRC patients diagnosed during asymptomatic screening colonoscopies between SH and UH (16.9% vs. 22.5%).

**Table 3:** Estimated OR and 95% CI based on multivariable GLM. N = 284 after excluding age < 50 y and < 80 y and missing values OR, odds ratio; CI, confidence interval; GLM, generalized linear model

Variable	Levels	OR (95% CI)	P-value
Age (y)	Every 1 y increase in age	1.00 (0.96-1.05)	0.8501
Race	Black/AA vs. White/EA	0.97 (0.16-5.85)	0.9698
	Other vs. White/EA	1.45 (0.25-8.44)	0.6820
Insurance	Medicare vs. Commercial	0.66 (0.22-1.99)	0.4569
	Medicaid vs. Commercial	0.70 (0.26 - 1.90)	0.4799
	Self-pay vs. Commercial	1.40 ( 0.53-3.77)	0.5117
Symptoms	Yes vs. No	8.04 (1.85-35.01)	0.0055
Previous colonoscopy age 50- 80 y	Yes vs. No	0.53 (0.19-1.50)	0.2331
Institution	SH vs. UH	0.48 (0.08-2.93)	0.5117

## Discussion

This collaborative chart review study combining data from two hospitals serving two disparate populations, was conducted with the goal of developing an approach towards disentangling the complex socioeconomic and biological factors contributing to racial CRC disparities. Given the patchwork nature and stringent privacy regulations in the US healthcare system, it is difficult to access data on these multiple variables from multiple centers. Furthermore, careful manual curation of the medical records revealed multiple inaccuracies in the categorization of symptoms, when based on the billing codes for screening vs. diagnostic colonoscopies. These inaccuracies are possibly related to differences in patient out-of-pocket costs between the screening vs. diagnostic colonoscopies [10].

 To identify variables associated with newly diagnosed patients presenting with metastatic stage 4 CRC, we included variables that we had previously included in our analysis of adenoma detection in initial screening colonoscopies [11]. Additional variables included in this study, were symptoms at diagnosis, history of previous colonoscopy (age 50-80 y), CRC location (right colon, left colon, rectum), and institution (SH vs. UH). Comparison of stage 0-3 CRC and stage 4 CRC subjects, revealed significant differences in insurance, symptoms, previous colonoscopy, and institution. The percentage of newly diagnosed CRC presenting with metastatic disease was 21.7% at the UH, 12.7% at the SH, compared to the overall US average of 20% [12]. Race, ethnicity, diabetes mellitus, and smoking status were not significantly different on univariate analyses. However, symptoms at diagnosis were the only variable significantly associated with stage 4 CRC, in a GLM controlling for age, race, insurance, previous colonoscopy, and institution. 

Approximately 80% of the CRC diagnoses presented with symptoms at both SH and UH, without any significant difference between the two institutions. In the combined SH and UH dataset, Black/AA race, insurance status, and institution are correlated with each other. A retrospective cohort study using veterans affairs (VA) national clinical performance and clinical data indicated that many racial/ethnic disparities reported in non-VA populations do not exist in the VA [13], where healthcare access is not correlated with race.

Limitations of this study relate to the small sample size from only two institutions. In this retrospective study we could not ascertain for many of the subjects, the time interval between the prior colonoscopy and the colonoscopy diagnosing the CRC. This made it difficult to determine whether the interval between the two colonoscopies was within the current recommended surveillance guidelines [8]. The Black/AA population served by the UH is primarily Afro-Caribbean in origin [14], and may not be representative of other Black/AA populations in the US with respect to adherence to CRC screening [15]. Insurance status was selected as the major socioeconomic factor related to access to quality healthcare in this study because it is invariably recorded in the medical records. However, there is tremendous heterogeneity among various commercial healthcare policies with respect to access to physician practices and hospitals, and to out-of-pocket costs. It is possible that the out-of- pocket costs for an outpatient colonoscopy for some of the commercial insurance policies accepted at the SH, exceed the out-of-pocket costs for uninsured patients at the UH. 

Taken together our results underscore the importance of screening patients before they become symptomatic. It is also important to note that the median age of initial CRC screening at both SH and UH was 55 y [11], five years after the previous USPSTF recommendation for initiating CRC screening at age 50 y, the USPSTF recommended age during the period of the study (2010-2018). Data on the percentage of US adults age who have initiated CRC screening at the USPSTF recommended age are not available, but is likely to be much lower than the 69.7% up to date with CRC screening rate for patients age 50-75 y. Expanding initiation of CRC screening at the USPSTF recommended age, recently lowered from 50 to 45 y [16], could potentially increase the proportion of CRC diagnosed during asymptomatic screening colonoscopies and reduce the proportion of metastatic stage 4 CRC diagnosed at presentation.

## Conclusions

In a combined sporadic CRC (2010-2018) dataset collected from two institutions serving two disparate populations, a higher proportion of newly diagnosed CRC patients presented with metastatic stage 4 CRC at the UH than the SH. However, multivariable analysis only identified the presence of symptoms at CRC diagnosis as significantly associated with stage 4 CRC, while controlling for age, race, insurance, previous colonoscopy, and institution. The results underscore the importance of screening patients before they become symptomatic.
